# Multimodality Evaluation of Turner Syndrome With Right Ventricular Dilation and Partial Anomalous Pulmonary Venous Return

**DOI:** 10.7759/cureus.73955

**Published:** 2024-11-18

**Authors:** Manvita Tatavarthy, Darren C Tsang, Antone Tatooles, Tisha M Suboc, Karolina Marinescu

**Affiliations:** 1 Cardiology, Rush University Medical Center, Chicago, USA; 2 Cardiovascular and Thoracic Surgery, Rush University Medical Center, Chicago, USA

**Keywords:** cardiac computed tomography (cct), cardiac magnetic resonance imaging, congenital heart defect (chd), eisenmenger syndrome, right heart catheterization, right-to-left cardiac shunt, transthoracic and transesophageal echocardiography, turner’s syndrome

## Abstract

Turner syndrome is a rare chromosomal abnormality in women that is caused by a partial or complete loss of one X chromosome and is often associated with a spectrum of congenital cardiac abnormalities, including cardiac shunts.

A 27-year-old woman with Turner syndrome was also found to have right ventricular dilation, partial anomalous pulmonary venous return, and possible atrial septal defect. She was scheduled for elective surgical repair. However, a preoperative review of diagnostic imaging raised the specter of Eisenmenger syndrome, a highly morbid condition defined by secondary, severe pulmonary hypertension where shunt correction may potentiate acute hemodynamic collapse. Multimodality imaging and invasive hemodynamics were used to comprehensively evaluate shunt physiology and guide surgical correction.

## Introduction

Turner syndrome is a rare sex chromosomal abnormality in women, occurring in about 1 in 2,500 live births [1]. It is caused by a partial or complete loss of the X chromosome and is often diagnosed prenatally or in early childhood. Turner syndrome is also associated with congenital heart disease [2]. The most common cardiac manifestations are bicuspid aortic valve and coarctation of the aorta, seen in up to 30% and 12% of people living with Turner syndrome, respectively [3]. As such, current clinical practice guidelines recommend that patients diagnosed with Turner syndrome visit a cardiologist at least once in their lifetime for screening [4].

This case describes the diagnostic work-up of a woman with Turner syndrome found to have partial anomalous pulmonary venous return (PAPVR), a less commonly associated defect in which one or more pulmonary veins drain into a systemic vein or the right atrium (RA) rather than the left atrium. Like unrepaired atrial septal defects (ASDs), PAPVR is a condition that can lead to hemodynamically significant left-to-right shunting of blood as defined by a pulmonary to systemic flow ratio (Qp:Qs) greater than 1.8 [5]. If left untreated, patients with significant shunts may progressively develop right ventricular dilation, irreversible pulmonary hypertension, and clinical heart failure. A feared and late-stage complication is Eisenmenger syndrome, a highly morbid condition defined by severe pulmonary hypertension leading to shunt reversal, where blood flows right-to-left through the defect resulting in systemic hypoxemia [6]. Hence, the characterization of shunt physiology is paramount to informing treatment decisions and may necessitate multimodality imaging and invasive hemodynamics. This article was previously presented as a meeting abstract at the American College of Cardiology 2024 Scientific Session on April 6, 2024.

## Case presentation

**Figure 1:**
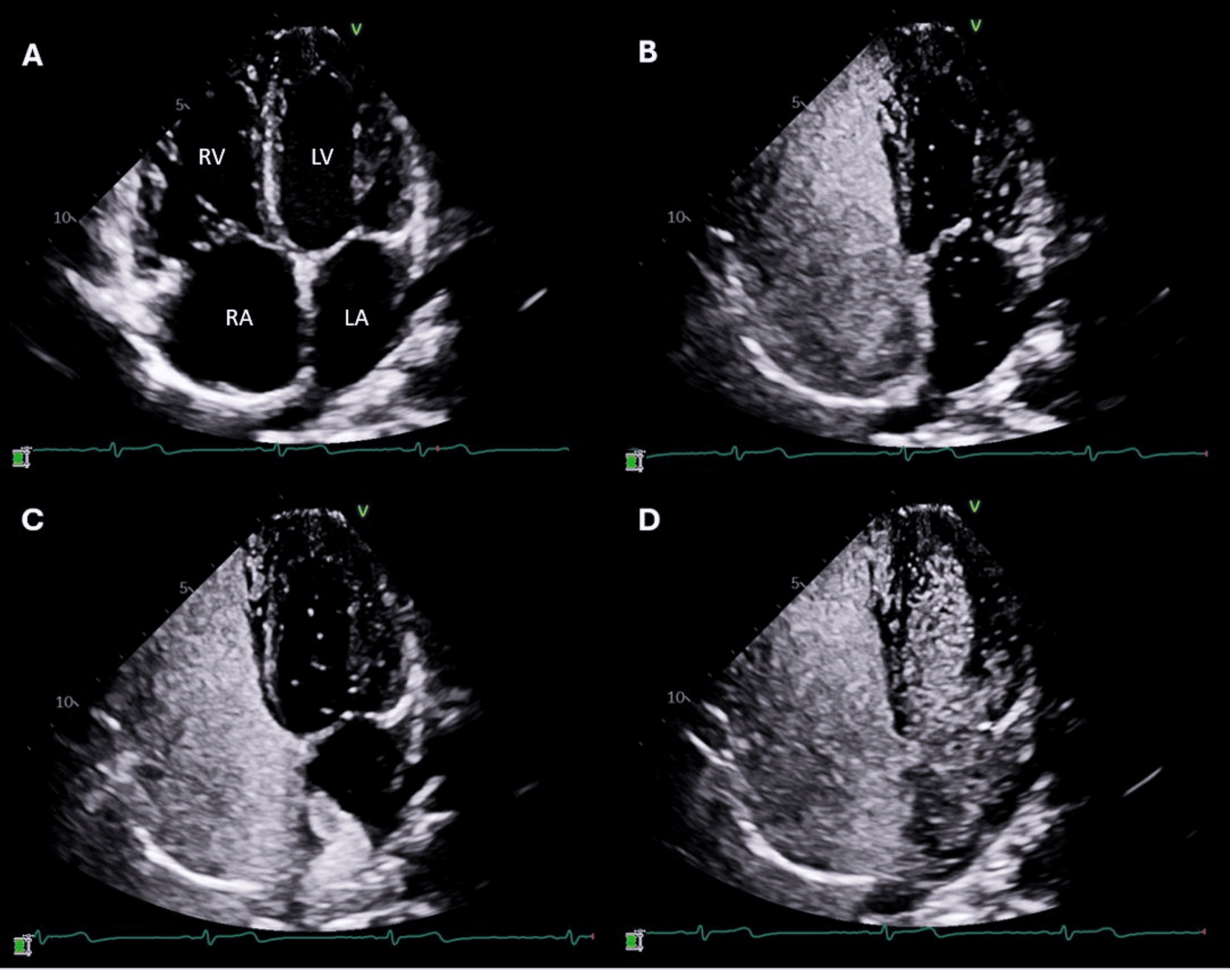
Positive agitated saline contrast study demonstrating a surge of bubbles from the direction of the right pulmonary vein after two cardiac cycles. (A) Apical four-chamber view demonstrating right atrial (RA) and right ventricular (RV) dilation. (B) Agitated saline contrast enters the right-sided chambers, and a few bubbles appear in the left atrium (LA). (C) Surge of bubbles entering the LA from the direction of the right pulmonary vein within two cardiac cycles. (D) Bubbles completely opacify the left-sided chambers, including the left ventricle (LV), by the third cardiac cycle.

**Figure 2:**
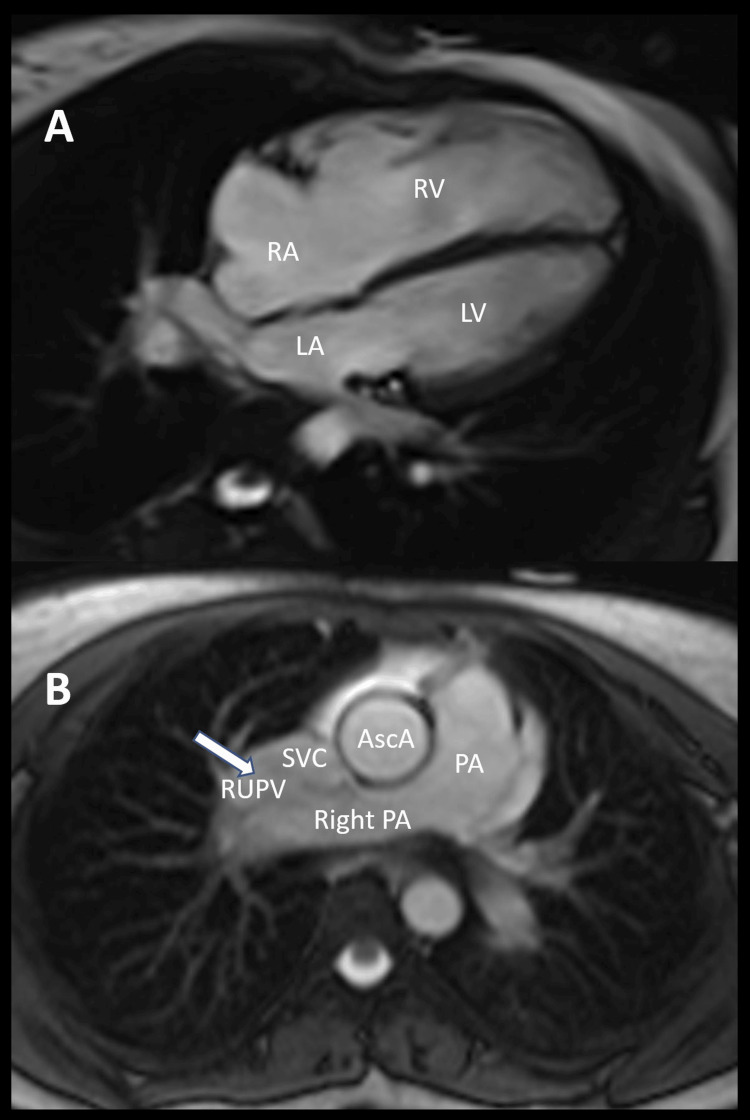
Cardiac MRI revealing partial anomalous pulmonary venous return. (A) Still image from a four-chamber cine showing significant right ventricular (RV) dilation with the right atrium (RA), left atrium (LA), and left ventricle (LV) labeled, respectively. (B) Axial image of partial anomalous pulmonary venous return (arrow) from the right upper pulmonary vein (RUPV) into the superior vena cava (SVC). Main pulmonary artery (PA) is dilated. Ascending aorta (AscA) and right pulmonary artery (PA) are labeled.

An asymptomatic 27-year-old woman with no past medical history presented to the gynecology outpatient clinic with irregular menses and premature ovarian failure. She underwent genetic testing and was diagnosed with Turner syndrome, as evidenced by a partial long-arm deletion of an X chromosome on peripheral blood lymphocyte karyotype analysis. According to screening guidelines, she was evaluated in a cardiology clinic for congenital cardiovascular defects. While sedentary, she denied exercise intolerance or shortness of breath. Her cardiac exam was unremarkable, with no extracardiac sounds. Physical manifestations of Turner syndrome, including short stature, webbed neck, digital abnormalities, and shield chest, were absent. Routine laboratory testing was unremarkable, including creatinine and hemoglobin levels within the normal ranges. Transthoracic echocardiogram (TTE) and cardiac MRI were subsequently performed. TTE showed normal left ventricular ejection fraction but with a dilated RA and right ventricle. The agitated saline bubble study demonstrated a very early and large shunt at rest without the Valsalva maneuver (Figure 1). Color Doppler interrogation did not reveal a shunt across the interatrial septum. The aortic valve was trileaflet, and the descending aorta was unremarkable. Cardiac MRI showed a severely dilated right ventricle with normal systolic function and findings suggestive of an anomalous right upper pulmonary vein draining into the superior vena cava (SVC) with an estimated Qp:Qs of 2.1 (Figure 2). An ASD could not be excluded by MRI. 

**Figure 3:**
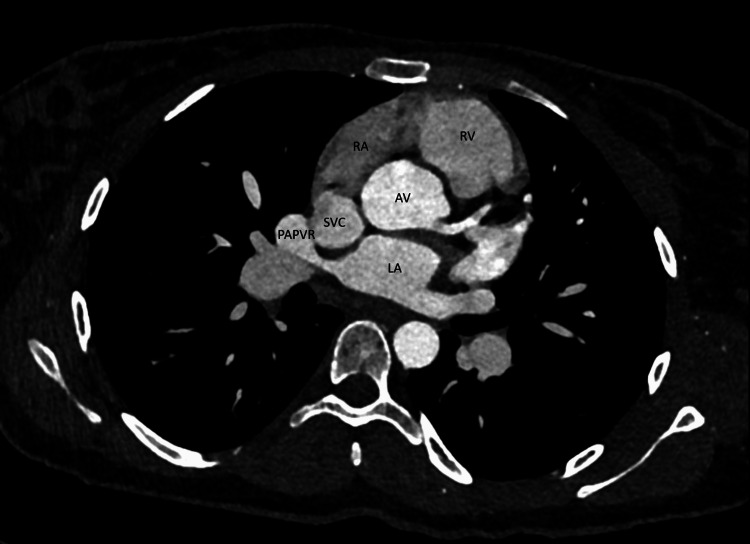
Cardiac CT demonstrating partial anomalous pulmonary venous return (PAPVR) of the right upper pulmonary vein draining into the superior vena cava. Axial image of PAPVR into the superior vena cava (SVC). Relevant adjacent cardiac structures such as the left atrium (LA), right atrium (RA), right ventricle (RV), and aortic valve (AV) are labeled, respectively.

Cardiac CT was next performed to further characterize the defect. It showed dilatation of the right-sided cardiac chambers (Figure 3). Right upper lobe PAPVR was observed with what appeared to be an associated sinus venosus ASD. The main pulmonary artery was also dilated suggestive of underlying pulmonary hypertension. The patient was subsequently evaluated by a cardiothoracic surgeon and scheduled for elective ASD and PAPVR repair.

Prior to surgery, a multidisciplinary review of the diagnostic imaging raised the specter of Eisenmenger’s syndrome. While cardiac MRI Qp:Qs greater than 1 is consistent with left-to-right shunting, marked right-to-left shunting was visualized on the TTE bubble study at rest and was suggestive of a significant RA-to-left atrium pressure gradient. She subsequently underwent preoperative right heart catheterization revealing normal right and left intracardiac filling pressures (Table 1). Shunt run demonstrated a significant step-up in oxygen saturation between the SVC and SVC/RA junction (75% to 89%), with a calculated Qp:Qs exceeding 2.5, indicating significant left-to-right shunt at the SVC/RA junction and confirming cardiac MRI findings.

**Table 1:** Right heart catheterization with shunt run study revealing normal cardiac filling pressures and left-to-right shunting at the junction of the superior vena cava and right atrium. Filling pressures are provided in systolic/diastolic where appropriate.

Location	Filling Pressures (mmHg)	Shunt Run (O_2_ Saturation)
Superior vena cava		75.0%
Superior vena cava and right atrial junction		89.0%
Right atrium	8/6	85.0%
Inferior vena cava	7/6	76.0%
Right ventricle	23/1	86.0%
Right pulmonary artery	16/7	89.0%
Left upper pulmonary artery (wedge position)	7	99.0% (arterial saturation)

**Figure 4:**
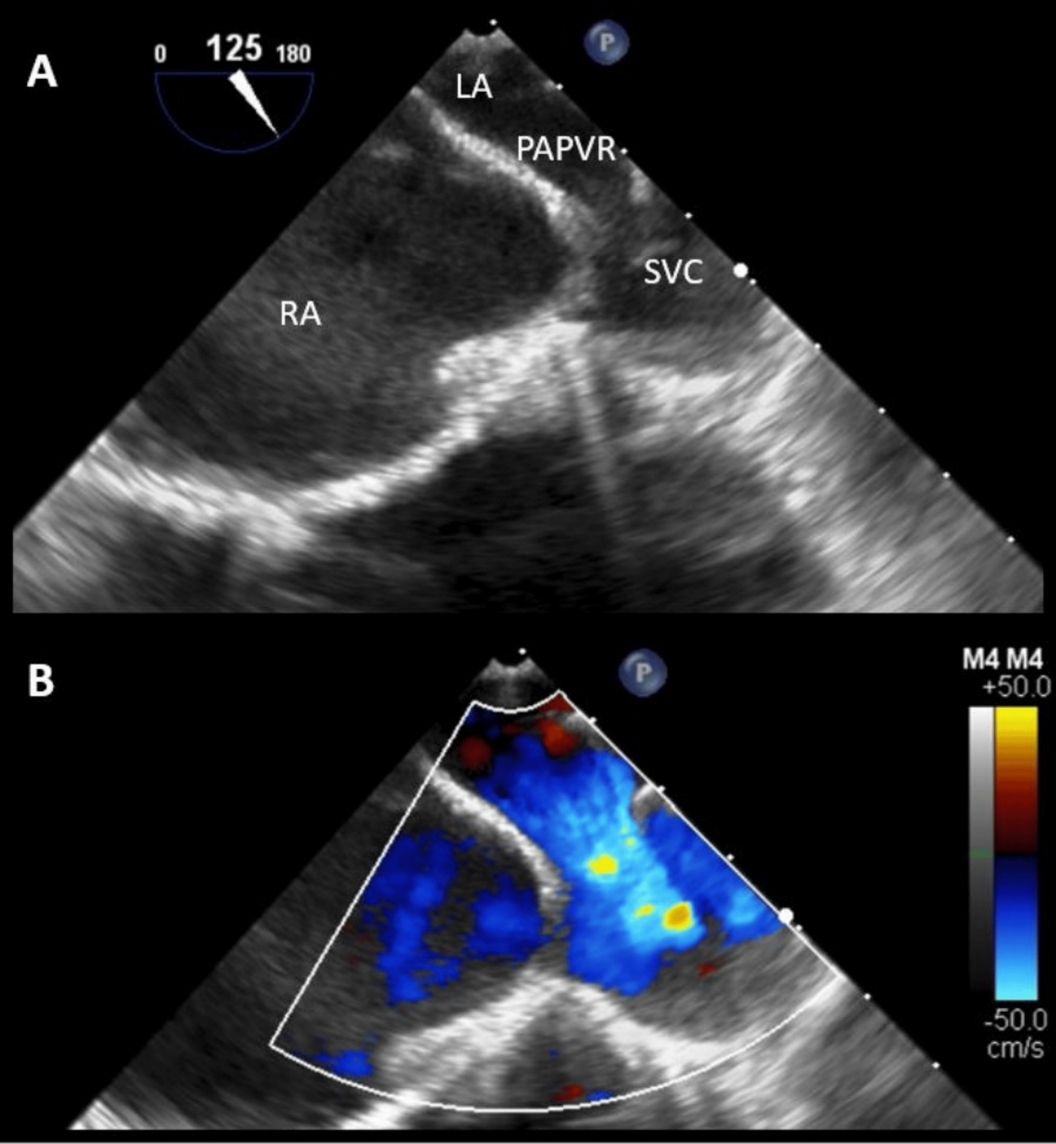
Perioperative transesophageal echocardiogram revealing right upper pulmonary vein insertion into the superior vena cava. (A) Modified mid-esophageal bicaval view at 125° with clockwise rotation. (B) Color Doppler image demonstrating left-to-right flow from the left atrium (LA), through partial anomalous pulmonary venous return (PAPVR), into the superior vena cava (SVC), and then the right atrium (RA).

The patient then underwent perioperative transesophageal echocardiography under general anesthesia, which showed PAPVR to the SVC with the anomalous vein inserting just proximal to the SVC/RA junction (Figure 4). No definitive ASD was visualized. Repeat agitated saline bubble study was negative while under positive-pressure ventilation. She subsequently underwent surgical repair of the defect via isolation of the right anomalous pulmonary vein baffled to the left atrium through a surgically created septal defect with pericardium patch repair, and closure of the SVC and RA with a bovine patch. Postoperative recovery was uneventful, and she was successfully discharged four days after surgery. Surveillance TTE at 6-month follow-up revealed her right-sided chambers had normalized.

## Discussion

PAPVR was previously thought to occur in patients with Turner syndrome rarely. In the current published literature, there are few case reports of PAPVR in Turner syndrome patients. One such report describes a 6-year-old girl with Turner syndrome, an aberrant subclavian artery, and PAPVR, which were diagnosed using cardiac CT [7]. Another case describes the work-up of an incidental lung shadow found on a routine CT of the chest in a woman with Turner syndrome in her late twenties, who was ultimately diagnosed with a pulmonary venous varix and PAPVR following echocardiography, CT pulmonary angiography, and cardiac catheterization [8].

Two recent studies have specifically evaluated the prevalence of PAPVR in patients with Turner syndrome [5,9]. One study showed a higher prevalence of PAPVR in their population when screened using cardiac MRI and TTE, 18%, compared to previously reported data ranging from 1.5% to 3.7% with TTE alone [5]. Another study identified a surprisingly high prevalence of PAPVR in Turner syndrome patients, 25%, by utilizing both CT angiography and MRI. The authors postulate that CT and MRI are more sensitive than echocardiography in identifying this lesion, which may have led to an underestimation of PAPVR in the past [9].

In our patient, the initial TTE agitated saline bubble study showed an early and marked surge of bubbles into the left atrium without the Valsalva maneuver. This raised the possibility of Eisenmenger syndrome, a condition that is defined by severe, secondary pulmonary hypertension in the setting of unrepaired congenital heart disease with resultant large anatomic shunts, leading to reversed or bidirectional shunting [6]. In these patients, ASD closure is harmful, as it can potentiate right ventricular failure and acute hemodynamic collapse [10]. Due to the possibility of this life-threatening complication, invasive hemodynamics were obtained. Evaluation with right heart catheterization demonstrated a left-to-right shunt, which was successfully corrected via surgical repair of this patient’s defect. We theorize that the initial shunt seen on TTE with bubble study was a dynamic, bidirectional shunt in which a predominantly left-to-right shunt transiently reversed with inspiration. Agitated saline injected into the venous system passed rapidly through the PAPVR via the SVC when intrathoracic pressure decreased and appeared in the left atrium within two cardiac cycles. During perioperative transesophageal echocardiogram under general anesthesia, repeated bubble study was negative as positive-pressure ventilation eliminated the right-to-left pressure gradient during respiration.

## Conclusions

PAPVR is an under-recognized cardiac anomaly associated with Turner syndrome, which may lead to significant shunting and right-sided volume overload if left untreated. This case highlights the importance of multimodality imaging and invasive hemodynamics in diagnostically challenging cases. The combination of cardiac imaging techniques such as echocardiography, MRI, CT, and catheterization allowed our treatment team to comprehensively characterize this patient’s congenital lesion so that she could undergo the appropriate definitive management.
